# Use and filling out of the child health booklet among beneficiaries of the Bolsa Família Program in Salvador-Bahia, Brazil: a cross-sectional study, 2023

**DOI:** 10.1590/S2237-96222024V33E2024498.EN

**Published:** 2024-11-11

**Authors:** Claudia Nery Teixeira Palombo, Márcia Maria Carneiro Oliveira, Maria Carolina Ortiz Whitaker, Ráren Paulo da Silva Araújo, Carolina de Jesus Santos, Mariana Cavalcante Brotas Passos, Clariana Vitória Ramos de Oliveira, Ednir Assis Souza

**Affiliations:** 1 Universidade Federal da Bahia, Escola de Enfermagem, Salvador, BA, Brasil; 2 Universidade Federal da Bahia, Faculdade de Medicina da Bahia, Salvador, BA, Brasil; 3 Universidade de Nevada, Escola de Enfermagem de Las Vegas, Nevada, Estados Unidos

**Keywords:** Child Health, Child Development, Primary Health Care, Social Programs, Cross-Sectional Studies, Salud Infantil, Desarrollo Infantil, Atención Primaria de Salud, Programas Sociales, Estudios Transversales

## Abstract

**Objective:**

To compare child health booklet (CHB) use and filling out among mothers who were or were not *Bolsa Família* Program (BFP) beneficiaries.

**Methods:**

Cross-sectional study with mothers of children <6years attending health centers in Salvador, Bahia, Brazil, between January-February/2023. The CHB was evaluated regarding its use (having a CHB, having it in hand, and having read it) and filling out (growth curves, development monitoring forms, and vaccination completeness). Descriptive statistics and the chi-square test were used.

**Results:**

Of the 411 study participants, 66% were BFP beneficiaries. Significant differences were found between the groups regarding CHB use: having a CHB (p < 0.001), having it in hand (p = 0.037), and having read it (p < 0.001). Significant difference in vaccination completeness was found (p = 0.005).

**Conclusion:**

There was a significant difference in CHB use and vaccination completeness when comparing mothers who were BFP beneficiaries and those who were not.

## Study contributions

**Main results:**

The Child Health Booklet achieved a higher proportion of use and filling out among the group of mothers benefiting from the *Bolsa Família* Program. Despite this, use and filling out of the booklet are still below expectations.

**Implications for services:**

Completely filling out the Child Health Booklet must be part of the routine monitoring of the growth and development of all children, especially those benefited by the *Bolsa Família* Program, as they are the most vulnerable group.

**Perspectives:**

Continuing education actions and the obligation to fill out the booklet as a condition of being benefited by the *Bolsa Família* Program can contribute to the effective monitoring of all children.

## Resumen

**Objetivo:**

Comparar uso y registro del carné de control pediátrico (CCP) entre madres beneficiarias y no beneficiarias del Programa Bolsa Familia (PBF).

**Métodos:**

Estudio transversal entre enero y febrero del 2023 con madres de niños < 6años, en unidades de salud de Salvador-Bahía. Se evaluó CSN en términos de uso (tener el carné, tenerlo en mano y haberlo leído) y registro (curvas de crecimiento, formularios del desarrollo y vacunación). Se utilizó estadística descriptiva y prueba de chi-cuadrado.

**Resultados:**

De los 411 participantes, el 66% fueron del PBF. Hubo diferencia significativa entre los grupos respecto al uso del CCP: tenerlo (p < 0,001), concurrir con él (p = 0,037) haberlo leído (p < 0,001). Respecto al registro, hubo diferencia significativa sólo en vacunación (p = 0,005).

**Conclusión:**

Hubo diferencia significativa en el uso y cumplimiento de la vacunación, comparando madres beneficiarias y no beneficiarias del PBF.

## INTRODUCTION

The *Bolsa Família* Program is an inter-ministerial political strategy that focuses on addressing social inequalities and combating hunger in Brazil. Created more than 20 years ago, it is considered one of the largest income transfer programs in the world, providing assistance to approximately 14 million families in all Brazilian municipalities.^1^

The Program is structured around three main pillars: income transfer itself; requirements involving access to healthcare; and education, social services and complementary programs. The second pillar, which refers to requirements involving access to healthcare, includes monitoring child growth and development and the vaccination schedule,^2^ ^)^ which must be duly recorded in the child health booklet.^3^

The child health booklet must be provided for every child born in public or private maternity hospitals at the time of hospital discharge and must be filled out by the health professionals who monitor the child, as recommended by the National Policy for Comprehensive Child Health Care.^3^ In 2018, the Ministry of Health presented the child health booklet as an advanced version of the Booklet distributed with effect from 2005,^4^ which had already succeeded the Child Health Card used with effect from the 1970s.^5^ In this updated 2018 version, fields were included to allow to education and social service professionals to make attendance records, in order to promote more effective integration in child monitoring between these three areas.^4^ ^)^

Booklets for monitoring children’s health are used in more than 160 countries around the world, and evidence indicates that their adequate use and filling out contribute to improvements in children’s health, with positive impacts on reducing child morbidity and mortality.^6^ However, in Brazil it can be seen that there are still important flaws in its filling out, especially regarding growth charts and child development monitoring sheets.^7^ ^),(^ ^8^ A scoping review, which systematically mapped studies involving the child health booklet in Brazil, identified weaknesses in filling out all the items in the Booklet in the 129 articles evaluated, except regarding vaccination, but did not find consensus regarding factors associated with filling out the Booklet items^.(^ ^9^

As far as we know, there are still no studies that investigate the relationship between the health requirements of the *Bolsa Família* Program and the child health booklet. Therefore, this study will be able to expand understanding of this topic and offer support for strategies that seek to effectively use and fill out the child health booklet in health services. The hypothesis was that there would be a higher proportion of use and filling out of the child health booklet among mothers benefiting from the *Bolsa Família* Program. 

The objective of this study was therefore to compare the use and filling out of the child health booklet among mothers who were or were not Bolsa Família Program beneficiaries.

## METHODS

*Study design and location*

This is a cross-sectional study, following the guidelines provided in the document entitled Strengthening the Reporting of Observational Studies in Epidemiology (STROBE).^10^ ^)^

The study was conducted in 27 family health centers, distributed over the 12 health districts in the city of Salvador-BA. The municipality has 155 primary health care (PHC) centers, including 46 basic health centers and 109 family health centers, with almost 60% PHC coverage.^11^ The city is located on the northeast coast of Brazil, has an estimated population of around 2,418,005 inhabitants, of whom 213,765 are children under 6 years old, whereby it has the fourth largest population among Brazilian municipalities and the largest in the Northeast region of the country. Salvador covers an area of 693.4 km², with an estimated population density of 3,486.96 inhabitants/km^2^. The Municipal Human Development Index is 0.759,^11^ but there are areas with great social inequalities, marked by poverty and the dominance of criminal drug trafficking factions, places where the health centers in this study are located. Despite this, the urban area, tourism and commerce are well developed. 

*Study population and sample*

The population was made up of mothers and their respective children under 6 years of age. The sample selection was proportional to the number of children per age group registered in each health district. The calculation took into consideration the proportion of children with inadequate feeding practices (p = 50%),^12^ in relation to the larger project; the population of children under 6 years old registered in the health districts of Salvador, by age group, according to Brazilian Institute of Geography and Statistics (IBGE) data for the year 2010,^13^ which was 199,489, since, during the project preparation period, the IBGE report for the year 2022 was not yet available; a 95% confidence level, 5% margin of error, as well as losses of 20%. Thus, the calculation indicated the need for a sample of 388 children. 

*Inclusion and exclusion criteria*

Children up to 6 years of age were included, accompanied by their biological mothers, excluding those with chronic or neurological diseases, as they require greater health care and could have more frequent access to health services, in additional to health professionals consequently paying greater attention when filling out the child health booklet. In the case of mothers accompanied by more than one child within this age range, the interview was based on the youngest child, in order to minimize possible memory errors for mothers with more than one child.

*Data collection*

Data collection occurred simultaneously in all health districts in the municipality. Mothers with their respective children were approached at the health centers and invited to participate in the research. 

The interviews were conducted by 12 undergraduate students from health courses (medicine, nursing, physiotherapy and nutrition), duly trained, who used a private room so as to guarantee the privacy of the participants. The interviews lasted approximately 40 minutes.

The mothers were interviewed using a specific pre-tested questionnaire, collecting data on maternal characteristics: age (in completed years), race/skin color (Asian, White, Indigenous, mixed race and Black), schooling in years (< 9 years , > 9 years), works away from home (yes/no) and whether the mother was a *Bolsa Família* Program beneficiary. Child characteristics were also collected: age group (< 6 months/from 6 months to 2 years/from 2 to 6 years), sex (male/female), low birth weight (below 2,500 g) (yes/no); and attends daycare (yes/no).

Use of the child health booklet was assessed through the following questions: *Do you have a Health Ministry child health booklet?* (yes/no); *Do you have your child health booklet with you?* (yes/no); *Have you read the child health booklet?* (fully/partially/never/I don’t have one). 

Child health booklet filling out was assessed by directly checking the recording of weight and height curves (yes/no/incomplete); records on the child development monitoring sheets (yes/no/incomplete); and, vaccination completeness (yes/no). Booklets without one of the items relating to each topic were considered incomplete, respecting the child’s age and follow-up calendar, as recommended by the Ministry of Health.^14^

*Data analysis and processing*

The database was built based on the Google Forms data collection form used, which enables the information collected to be stored on an Excel spreadsheet. All answers obtained were assessed for information consistency. Data processing and analysis were carried out using SPSS software, version 24.0. We used simple frequency and percentage of nominal variables and continuous variables. Mean and standard deviation were used, due to normality using the Kolmogorov-Smirnov test. In order to compare the group in which mothers were *Bolsa Família* Program beneficiaries with the group in which mothers were not Program beneficiaries, we used Pearson’s chi-square test and Fisher’s exact test, taking a significance level of <5%.

Quality control measures included the use of pre-tested and standardized instruments; the preparation of a manual with detailed guidelines for carrying out the interviews and filling out the form; and thorough training of the entire team for data collection and fieldwork supervision. Furthermore, a random sample of 5% of the interviews was repeated by the researchers themselves (via telephone) to verify the quality and veracity of the information.

*Ethical aspects*

The project was submitted to and approved by the Research Ethics Committee of the *Escola de Enfermagem da Universidade Federal da Bahi*a (File No.: 5.800.539). All participants signed a Free and Informed Consent form. The current standards for research involving human beings were respected, according to National Health Council Resolution No. 466/2012, via the National Research Ethics Committee, in addition to the principles of autonomy, justice, equity, beneficence and nonmaleficence.

## RESULTS

Of a total of 757 mothers approached at health units, 201 refused to participate in the research and 145 did not meet the inclusion criteria: nine had neurological diseases and 136 were not biological mothers. Thus, 411 pairs of mothers and children participated in the study, of whom 65.9% (n = 271) were *Bolsa Família* Program beneficiaries. 

Table 1 shows that the mothers’ mean age was 31 years (SD + 7 years), almost all mothers were Black (94.4%), more than a third (32.1%) had only elementary education (9 years of study) and the majority did not work away from home (63.9%), with a statistically significant difference in these characteristics between the groups of mothers who were and were not *Bolsa Família* Program beneficiaries. As for the children, 63% were between 2 and 6 years old and more than half (53.7%) did not attend daycare or preschool.

**Table 1:** Distribution of the characteristics of mothers and children who were and were not *Bolsa Família* Program beneficiaries, Salvador, Bahia, Brazil, 2023 (n = 411)

Variables		***Bolsa Família* Program Beneficiaries**		p-value^a^
	Total	No (n = 140)	Yes (n = 271)	
	n (%)	n (%)	n (%)	
Maternal characteristics				
Mothers’ age (mean; SD)		(31; +7)	(31; +7)	
Race/skin color				0,011
Asian	4 (1,0)	01 (25,0)	03 (75,0)	
White	18 (4,4)	11 (61,1)	07 (38,9)	
Indigenous	1 (0,2)	01(100,0)	-	
Mixed race	195 (47,4)	74 (37,9)	121 (62,1)	
Black	193 (47,0)	53 (27,5)	140 (72,5)	
Schooling (years)^b^				< 0,001
< 9	132 (32,4)	20 (15,2)	112 (84,8)	
> 9	278 (67,6)	120 (43,2)	158 (56,8)	
Works away from home				< 0,001
Yes	148 (36,0)	71 (48,0)	77 (52,0)	
No	263 (64,0)	69 (26,2)	194 (73,8)	
Child characteristics				
Child’s age				0,011
< 6 months	44 (10,7)	22 (50,0)	22 (50,0)	
6 months - 2 years	107 (26,0)	42 (39,3)	65 (60,7)	
> 2 years - 6 years	260 (63,3)	76 (29,2)	184 (70,8)	
Child’s sex				0,034
Male	206 (50,1)	60 (29,1)	146 (70,9)	
Female	205 (49,9)	80 (39,0)	125 (61,0)	
Low birth weight^b^				0,695
Yes	45 (10,9)	11 (24,5)	34 (75,5)	
No	356 (86,6)	124 (34,8)	232 (65,2)	
Attends daycare/preschool				0,107
Yes	190 (46,2)	57 (30,0)	133 (70,0)	
No	221 (53,8)	83 (37,6)	138 (62,4)	

a) Pearson’s chi-square test; Fisher’s exact test; b) It was not possible to obtain information for the entire sample.

Table 2 presents the comparison of characteristics between groups of mothers who were and were not *Bolsa Família* Program beneficiaries, according to use and filling out of the child health booklet. A higher proportion of Booklet use was found among mothers benefiting from the Program, with a significant difference: 70% had a Booklet (p < 0.001), 63.1% had a Booklet with them (p = 0.037) and 65.8% had read the Booklet completely (p < 0.001). Regarding filling out, of the 282 mothers who had the Booklets with them at the time of the interview, only 32.9% of the growth charts were completely filled out (p = 0.579); 25.2% of the child development sheets were filled out (p = 0.101); and the vaccination schedule was complete in 85.8% of them (p = 0.005).

**Table 2:** Distribution of the variables relating to use and filling out of the child health booklet of mothers and children who were and were not *Bolsa Família* Program beneficiaries, Salvador, Bahia, Brazil, 2023

Variables		***Bolsa Família* Program Beneficiaries**		p-value^a^
	Total	No (n = 140)	Yes (n = 271)	
	n (%)	n (%)	n (%)	
Do you have a Health Ministry child health booklet?				< 0,001
Yes	337 (82,0)	101 (30,0)	236 (70,0)	
No	74 (28,0)	39 (52,7)	35 (47,3)	
Do you have your child health booklet with you?				0,037
Yes	282 (68,6)	104 (36,9)	178 (63,1)	
No	129 (31,4)	36 (27,9)	93 (72,1)	
Have you read the child health booklet?				< 0,001
Fully	73 (17,8)	25 (34,2)	48 (65,8)	
Partially	203 (49,4)	71 (35,0)	132 (65,0)	
No	106 (25,8)	25 (23,6)	81 (76,4)	
I don’t have one	29 (7,0)	19 (65,5)	10 (34,5)	
Growth charts filled out^b^				0,579
Yes	93 (32,9)	33 (35,5)	60 (64,5)	
No	74 (26,2)	31 (41,9)	43 (58,1)	
Incomplete	115 (27,9)	40 (34,8)	75 (65,2)	
Child development sheet filled out^b^				0,101
Yes	71 (25,2)	24 (33,8)	47 (66,2)	
No	113 (40,1)	50 (44,2)	63 (55,8)	
Incomplete	98 (34,7)	30 (30,6)	68 (69,4)	
Vaccination schedule completeness^b^				0,005
Yes	242 (85,8)	80 (33,1)	162 (66,9)	
No	40 (14,2)	24 (60,0)	16 (40,0)	

a) Pearson’s chi-square test; b) Only the booklets that were with the mothers at the time of the interview (n = 282).

Although there was no significant difference, assessment of the filling out of the child health booklet by age group, among children benefiting from the Bolsa Família Program, showed a higher proportion of filling out of the growth charts (57.1%), child development monitoring sheets (35, 7%) and vaccination completeness (92.7%) among children under 6 months old, as shown in Table 3.

**Table 3:** Distribution of children beneficiaries of the *Bolsa Família* Program who had the child health booklet, with them at the time of the interview, by age and booklet filling out, Salvador, Bahia, Brazil, 2023

Age group	Total n	Growth chart filled out				Child development sheet filled out				Vaccination schedule completeness		
		Yes	No	Partial	p-value^a^	Yes	No	Partial	p-value^a^	Yes	No	p-value^a^
		n (%)	n (%)	n (%)		n (%)	n (%)	n (%)		n (%)	n (%)	
< 6 months	14 (7,9)	08 (57,1)	04 (28,6)	02 (14,3)	0,137	5 (35,7)	5 (35,7)	4 (28,6)	0,927	13 (92,7)	01 (7,1)	0,927
6 months a < 2 years	46 (25,8)	16 (34,8)	13 (28,2)	17 (37,0)		12 (26,1)	16 (34,8)	18 (39,1)		42 (91,3)	04 (8,7)	
> 2 years a 6 years	118 (66,3)	36 (30,5)	26 (22,0)	56 (47,5)		30 (25,4)	42 (35,6)	45(39,0)		107 (90,7)	11 (9,3)	
Total	178 (100,0)	60 (33,7)	43 (24,2)	75 (42,1)		47 (26,4)	63 (35,4)	68 (38,2)		162 (91,0)	16 (9,0)	

a) Pearson’s chi-square test; Fisher’s exact test.

## DISCUSSION

The results of this study showed that there was a higher proportion of use of the child health booklet among the group of mothers who benefited from the *Bolsa Família* Program, with a statistically significant difference when compared to the group of mothers who were not beneficiaries. Regarding the filling out of the booklets, there was no significant difference between the groups, but the results revealed that the growth charts and the child development monitoring sheets had a higher proportion of records among the group of mothers benefiting from the Program, with double the vaccination completeness when compared to the group of mothers who did not receive the benefit. Among the *Bolsa Família* Program beneficiaries, the highest proportion of filling out of the child health booklet was concentrated in the zero to 6 months age group, although there was no statistical significance.

The findings suggest that the group of mothers benefiting from the *Bolsa Família* Program may be more committed to using the Booklet, and this has significant implications for the promotion of child health,^6^ ^)^ but that, unfortunately, health professionals have not yet incorporated filling out the booklet into their practice as a tool for monitoring children’s health. 

There are literature reviews, which included studies from the 1990s to 2022, on the use of the child health booklet in the Brazilian context,^7^ ^),(^ ^9^ ^),(^ ^15^ that corroborate the results obtained, when they also point to underuse of the child health booklet by health professionals, being justified by health service infrastructure and operational problems, such as lack of time and training of professionals, lack of equipment for child anthropometry and forms for recording information.^16^ It is believed that these aspects affect adequate use of the Booklet, and understanding them is crucial for proposing strategies to address these problems.

Additionally, as an aggravating factor for low adherence to filling out growth charts and child development monitoring sheets among both groups of mothers, the Ministry of Health has not distributed the child health booklet since 2020, which further discourages its use by health professionals.^16^ This situation disregards the recommendations of the Child and Adolescent Statute on protection, guarantee of health and social responsibility in relation to children.^17^

It is our understanding that the health requirements recommended by the *Bolsa Família* Program could encourage greater use and filling out of the child health booklet, as they promote the active participation of families in health services, encouraging parental responsibility, as well as providing more regular monitoring of children in health services, facilitating access to preventive care and health promotion in early childhood for families facing greater social vulnerability.^18^

In fact, the results are encouraging when comparing mothers who were or were not *Bolsa Família* Program beneficiaries, showing that among the former there was greater use and filling out of the Booklets, in agreement with the hypothesis raised by this study. However, this proportion is still lower than expected, considering that this occurred in only approximately one third of the sample. A scoping review, which evaluated Brazilian studies from the 1990s to 2022, showed that filling out of growth charts can vary between 8.9% and 100%; that of child development monitoring sheets varies between 0% and 72.7%; and variation in vaccination completeness is between 91.8% and 100%.^9^

The existence of requirements to be fulfilled as established by the *Bolsa Família* Program aims to favor access to basic social rights regarding health and education, with a view to breaking the intergenerational cycle of poverty in beneficiary families.^19^ In this sense, it is worth highlighting the sociodemographic profile of the mothers who participated in the study. Young mixed race and Black mothers are the most vulnerable in Brazilian society, as they experience race, gender and class inequalities, as well as educational and income disadvantages, live in precarious housing conditions and, at the same time, have more children and fewer fixed partners.^20^ The direct relationship between extreme social vulnerability and the impacts on health and access to services is a fact, with negative consequences for the care of children. 

A study conducted with a cohort of 100 million Brazilians registered on the Federal Government’s Single Registry for Social Programs (*Cadastro Único para Programas Sociais do Governo Federal* - CadÚnico) analyzed 6,309,366 children under 5 years old between 2006 and 2015.^21^ The results showed that the *Bolsa Família* Program had a positive impact on improved health, through increased purchasing power, improved access to early childhood education and adherence to health programs for children, with a 17% reduction in the likelihood of deaths.^21^

The *Bolsa Família* Program places emphasis on the provision of actions that are already part of the child health care line in PHC services.^22^ ^)^ However, it can be seen that these actions end up being limited to merely administrative tasks, filling out spreadsheets, without there being, in most cases, assessments made by health professionals that can guarantee comprehensive health care for these children.^18^

A study that sought to understand the narratives of PHC professionals in the city of Rio de Janeiro, between 2018 and 2019, identified that, in practice, monitoring the health requirements of the Bolsa Família Program is reduced to “weighing and measuring” children, without nutritional diagnosis necessarily being performed to identify cases at risk and enable timely interventions.^18^ The bureaucratic view towards these health requirements is a cause for concern, i.e. just to ensure that families do not lose the benefit and that the *Bolsa Família* Program management targets are met, since the child being taken to the health center to enable Program data to be collected should be an opportunity for guidance, health education, assessment of the child and filling out of the child health booklet. However, it can be seen that little value is given to this opportunity when children are taken to their health centers.^23^

On the other hand, it is crucial to advance with health service reorganization and with the engagement and awareness of health professionals, to ensure that health and social interventions are offered in an integrated manner and with excellence for all families with very young children.^3^ ^),(^ ^19^ This implies promoting a comprehensive approach, which considers not only immediate health needs, but also social and economic issues that impact the well-being of families, integrating sectors such as health, education, social services and community development.^3^

This study was conducted at a single time and place, limiting the establishment of causal relationships in a temporal sequence, which may be a limitation. However, the study presents data that has been little explored on the use and filling out of the Booklet and its relationship with the *Bolsa Família* Program.

We conclude that *Bolsa Família* Program beneficiaries show greater use and filling out of the child health booklet. However, it is important to highlight that such filling out does not yet fully meet the recommendations of national health bodies. In view of this, it is necessary to implement ongoing in-service education actions, awareness raising and empowerment of families, such as awareness campaigns and health education, including educational materials, lectures and activities in health centers, in addition to encouraging co-responsibility in the health service territories, via community leaders. These measures are essential so that the Booklet can effectively play its role as an instrument for monitoring the growth and development of all children, thus promoting a significant improvement in child health indicators.
