# Supplementary material for: Use and filling out of the child health booklet among beneficiaries of the Bolsa Família Program in Salvador-Bahia, Brazil: a cross-sectional study, 2023
Source: Epidemiol Serv Saude. 2024 Nov 11;33:e2024498. doi: 10.1590/S2237-96222024V33E2024498.EN (PMC11554293; doi:10.1590/S2237-96222024V33E2024498.EN)
Supplement: Translation

# Translation: Uso e preenchimento da caderneta da criança entre beneficiários do Programa Bolsa Família de Salvador, Bahia: um estudo transversal, 2023

**DOI:** 10.1590/S2237-96222024V33E2024498.PT

**Authors:** Claudia Nery Teixeira Palombo, Márcia Maria Carneiro Oliveira, Maria Carolina Ortiz Whitaker, Ráren Paulo da Silva Araújo, Carolina de Jesus Santos, Mariana Cavalcante Brotas Passos, Clariana Vitória Ramos de Oliveira, Ednir Assis Souza

**Affiliations:** 1 Universidade Federal da Bahia, Escola de Enfermagem, Salvador, BA, Brasil; 2 Universidade Federal da Bahia, Faculdade de Medicina da Bahia, Salvador, BA, Brasil; 3 Universidade de Nevada, Escola de Enfermagem de Las Vegas, Nevada, Estados Unidos

**Keywords:** Saúde da Criança, Desenvolvimento Infantil, Atenção Primária à Saúde, Programas Sociais, Estudos Transversais

## Abstract

**Objetivo:**

Comparar uso e preenchimento da caderneta da criança entre mães beneficiárias ou não do Programa Bolsa Família.

**Métodos:**

Estudo transversal com mães de crianças menores de 6 anos em unidades de saúde de Salvador-BA, entre janeiro e fevereiro de 2023. A caderneta da criança foi avaliada quanto ao uso (ter a caderneta, estar com ela em mãos e tê-la lido) e preenchimento (curvas de crescimento, fichas de avaliação do desenvolvimento e completude vacinal). Utilizaram-se estatística descritiva e teste qui-quadrado, com nível de significância de 5%.

**Resultados:**

Das 411 participantes do estudo, 66% eram beneficiárias do programa. Houve diferença significativa entre os grupos quanto ao uso da caderneta: ter a caderneta (p < 0,001), estar com ela em mãos (p = 0,037) e tê-la lido (p < 0,001). Quanto ao preenchimento, houve diferença significativa apenas na completude vacinal (p = 0,005).

**Conclusão:**

Houve diferença significativa quanto ao uso e à completude vacinal, comparando-se mães beneficiárias e não beneficiárias do Programa Bolsa Família.

## Contribuições do estudo

**Principais resultados:**

A caderneta da criança alcançou maior proporção de uso e preenchimento no grupo de mães beneficiárias do Programa Bolsa Família. Apesar disso, o uso e o preenchimento da caderneta ainda estão aquém do esperado.

**Implicações para os serviços:**

O preenchimento completo da caderneta da criança deve integrar a rotina do acompanhamento do crescimento e desenvolvimento de todas as crianças, especialmente aquelas do Programa Bolsa Família, que constituem o grupo de maior vulnerabilidade.

**Perspectivas:**

Ações de educação permanente e a obrigatoriedade do preenchimento da caderneta como condicionalidade do Programa Bolsa Família podem contribuir para o efetivo acompanhamento de todas as crianças.

## INTRODUÇÃO

O Programa Bolsa Família é uma estratégia política interministerial que tem como foco o enfrentamento das desigualdades sociais e o combate à fome no Brasil. Criado há mais de 20 anos, é considerado um dos maiores programas de transferência de renda do mundo, abrangendo aproximadamente 14 milhões de famílias em todos os municípios brasileiros.^1^ ^)^

O programa estrutura-se em três principais eixos: a transferência de renda propriamente dita; as condicionalidades que envolvem o acesso à saúde; e a educação, a assistência social e os programas complementares. No segundo eixo, que se refere às condicionalidades que envolvem o acesso à saúde, encontra-se o acompanhamento do crescimento e desenvolvimento infantil e do calendário vacinal,^2^ que devem estar devidamente registrados na caderneta da criança.^3^

A caderneta da criança deve ser entregue à mãe de toda criança nascida em maternidade pública ou privada, no momento da alta hospitalar, e deve ser preenchida pelos profissionais que acompanham a criança, conforme preconiza a Política Nacional de Atenção Integral à Saúde da Criança.^3^ Em 2018, o Ministério da Saúde apresentou a caderneta da criança como uma evolução da versão distribuída em 2005,^4^ que já havia sucedido o Cartão da Criança dos anos 1970.^5^ Nesta versão atualizada de 2018, foram incluídos espaços destinados a permitir que profissionais da área da educação e do serviço social façam registros de atendimento, de modo a se promover uma integração mais eficaz no acompanhamento da criança entre essas três áreas.^4^

As cadernetas para o acompanhamento da saúde das crianças são utilizadas em mais de 160 países do mundo, e evidências apontam que seu uso e preenchimento adequados contribuem para melhorias na saúde das crianças, com impactos positivos na redução da morbimortalidade infantil.^6^ No entanto, no Brasil observa-se que ainda há falhas importantes em seu preenchimento, especialmente das curvas de crescimento e da ficha de avaliação do desenvolvimento infantil.^7^ ^),(^ ^8^ Uma revisão de escopo, que mapeou de forma sistemática os estudos que envolvem a caderneta da criança no país, identificou fragilidades no preenchimento de todos os itens da caderneta nos 129 artigos avaliados, exceto quanto à vacinação, mas não apontou consenso quanto aos fatores associados a esse preenchimento.^9^

Ao que se sabe, ainda não há estudos que investiguem a relação entre as condicionalidades de saúde do Programa Bolsa Família e a caderneta da criança. Assim, este estudo poderá ampliar o entendimento sobre essa temática e oferecer subsídios para estratégias que busquem efetivar o uso e o preenchimento da caderneta da criança nos serviços de saúde. A hipótese era de que haveria maior proporção de uso e preenchimento da caderneta da criança entre as mães beneficiárias do Programa Bolsa Família. 

Assim, o objetivo foi comparar uso e preenchimento da caderneta da criança entre mães beneficiárias e não beneficiárias do Programa Bolsa Família.

## MÉTODOS

*Delineamento e local do estudo*

Estudo transversal, orientado pela ferramenta Strengthening the Reporting of Observational Studies in Epidemiology (STROBE).^10^

O estudo foi conduzido em 27 unidades de saúde da família (USFs), distribuídas pelos 12 distritos sanitários do município de Salvador-BA. O município dispõe de 155 unidades da atenção primária à saúde (APS), sendo 46 unidades básicas de saúde (UBS) e 109 unidades de saúde da família, apresentando quase 60% de cobertura da APS.^11^ ^)^ A cidade está situada no litoral nordestino, tem uma população estimada em torno de 2.418.005 habitantes, dos quais 213.765 crianças menores de 6 anos de idade, registrando a quarta maior população entre os municípios brasileiros e a maior da região Nordeste. Salvador ocupa uma extensão territorial de 693,4 km², com uma densidade demográfica estimada de 3.486,96 hab./km^2^. O Índice de Desenvolvimento Humano Municipal é de 0,759,^11^ mas existem áreas com grandes desigualdades sociais, marcadas pela pobreza e pelo domínio de facções criminosas do tráfico de drogas, locais onde se distribuem as UBS do presente estudo. Apesar disso, a área urbana, o turismo e o comércio são bem desenvolvidos. 

*População de estudo e amostra*

A população foi composta por mães e seus respectivos filhos menores de 6 anos de idade. A seleção da amostra foi proporcional ao número de crianças por faixa etária, cadastradas em cada distrito sanitário. O cálculo considerou a proporção de crianças com práticas alimentares inadequadas (p = 50%),^12^ relativo ao projeto maior; a população de crianças menores de 6 anos cadastradas nos distritos sanitários de Salvador, por faixa etária, de acordo com dados do Instituto Brasileiro de Geografia e Estatística (IBGE) de 2010,^13^ que era de 199.489, pois, no período de elaboração do projeto, ainda não havia o relatório do IBGE de 2022; foram considerados nível de confiança de 95%, margem de erro de 5%, e um adicional de 20% de perdas. Assim, o cálculo indicou a necessidade de uma amostra de 388 crianças. 

*Critérios de inclusão e exclusão*

Foram incluídas crianças com até 6 anos de idade, acompanhadas por suas mães biológicas, sendo excluídas aquelas que tinham doenças crônicas ou neurológicas, pois essas necessitam de maior assistência de saúde e poderiam ter acesso aos serviços de saúde e mais frequência neles, havendo, consequentemente, maior atenção dos profissionais quanto ao preenchimento da caderneta da criança. Para as mães acompanhadas por mais de um filho dentro dessa faixa etária, a entrevista foi direcionada ao filho mais novo, para minimizar possíveis erros de memória das mães com mais de um filho.

*Coleta dos dados*

A coleta de dados ocorreu simultaneamente em todos os distritos sanitários do município. As mães, com seus respectivos filhos, foram abordadas nas unidades de saúde e convidadas a participar da pesquisa. 

As entrevistas foram conduzidas por 12 estudantes de graduação de cursos da área da saúde (medicina, enfermagem, fisioterapia e nutrição), devidamente treinados, que utilizaram uma sala reservada, garantindo a privacidade dos participantes. As entrevistas tiveram duração de aproximadamente 40 minutos.

As mães foram entrevistadas por formulário próprio, pré-testado, contendo dados sobre as características maternas: idade (em anos completos), raça/cor da pele (amarela, branca, indígena, parda e preta), escolaridade em anos (< 9 anos, > 9 anos), trabalho fora do lar (sim/não) e se era beneficiária do Programa Bolsa Família. Ainda foram coletadas características infantis: faixa etária (< 6 meses/de 6 meses a 2 anos/de 2 a 6 anos), sexo (masculino/feminino), baixo peso ao nascer (considerado peso inferior a 2.500 g) (sim/não); e frequenta creche (sim/não).

O uso da caderneta da criança foi avaliado pelas seguintes questões: *A senhora possui a caderneta da criança do Ministério da Saúde?* (sim/não); *A senhora está com a caderneta da criança em mãos?* (sim/não); *A senhora já leu a caderneta da criança?* (completamente/parcialmente/nunca li/não tenho). 

O preenchimento da caderneta da criança foi avaliado pela verificação direta quanto ao registro nas curvas de peso e estatura (sim/não/incompleto); registro nas fichas de avaliação do desenvolvimento infantil (sim/não/incompleto); e completude vacinal (sim/não). Foram consideradas incompletas as cadernetas com ausência de um dos itens referentes a cada tópico, respeitando-se a idade e o calendário de acompanhamento da criança, conforme recomendação do Ministério da Saúde.^14^

*Análise e processamento dos dados*

A construção do banco de dados deu-se a partir do formulário de coleta utilizado, o Google Forms, que permite o armazenamento das informações coletadas em uma planilha de Excel. Todas as respostas obtidas foram avaliadas quanto à consistência das informações, e o processamento e a análise dos dados foram realizados com uso do *software* SPSS, versão 24.0. Utilizaram-se frequência simples e percentual das variáveis nominais e das variáveis contínuas, média e desvio-padrão, devido à normalidade por teste de Kolmogorov-Smirnov. Para comparação entre os grupos de mães beneficiárias do Programa Bolsa Família e não beneficiárias, foram utilizados o teste qui-quadrado de Pearson e o teste exato de Fisher, com nível de significância menor que 5%.

As medidas de controle de qualidade incluíram o uso de instrumentos pré-testados e padronizados; a elaboração de manual com orientações detalhadas para a realização das entrevistas e preenchimento do formulário; e treinamento minucioso de toda a equipe para a coleta de dados e a supervisão do trabalho de campo. Além disso, uma amostra aleatória de 5% das entrevistas foi repetida pelas próprias pesquisadoras (via telefone), para se verificar a qualidade e a veracidade das informações.

*Aspectos éticos*

O projeto foi submetido e aprovado pelo Comitê de Ética em Pesquisa da Escola de Enfermagem da Universidade Federal da Bahia (processo: 5.800.539). Todos os participantes assinaram o Termo de Consentimento Livre e Esclarecido. Foram respeitadas as normas vigentes para pesquisa em seres humanos, segundo a Resolução nº 466/2012 do Ministério da Saúde e Conselho Nacional de Saúde, através da Comissão Nacional de Ética em Pesquisa, além dos princípios da autonomia, justiça, equidade, beneficência e não maleficência.

## RESULTADOS

De um total de 757 mães abordadas nas unidades de saúde, 201 se recusaram a participar da pesquisa e 145 não atendiam aos critérios de inclusão: nove tinham doenças neurológicas e 136 não eram mães biológicas. Assim, participaram do estudo 411 pares de mães e filhos, dos quais 65,9% (n = 271) eram de beneficiários do Programa Bolsa Família.

A Tabela 1 mostra que a média da idade das mães foi de 31 anos (dp + 7 anos), quase a totalidade das mães era da raça negra (94,4%), mais de um terço (32,1%) tinham apenas o ensino fundamental (9 anos de estudo) e a maioria não trabalhava fora do lar (63,9%), com diferença estatisticamente significativa dessas características entre os grupos de mães beneficiárias e não beneficiárias do Programa Bolsa Família. Quanto às crianças, 63% estavam na faixa etária de 2 a 6 anos de idade e mais da metade (53,7%) não frequentava creche ou pré-escola.

**Tabela 1:** Distribuição das características de mães e crianças beneficiárias e não beneficiárias do Programa Bolsa Família, Salvador, Bahia, 2023 (n = 411)

Variáveis		Beneficiárias do Programa Bolsa Família		
	Total	Não (n = 140)	Sim (n = 271)	p-valor^a^
	n (%)	n (%)	n (%)	
Características maternas				
Idade das mães (média; DP)		(31; +7)	(31; +7)	
Raça/cor da pele				0,011
Amarela	4 (1,0)	01 (25,0)	03 (75,0)	
Branca	18 (4,4)	11 (61,1)	07 (38,9)	
Indígena	1 (0,2)	01(100,0)	-	
Parda	195 (47,4)	74 (37,9)	121 (62,1)	
Preta	193 (47,0)	53 (27,5)	140 (72,5)	
Escolaridade (anos)^b^				< 0,001
< 9	132 (32,4)	20 (15,2)	112 (84,8)	
> 9	278 (67,6)	120 (43,2)	158 (56,8)	
Trabalha fora do lar				< 0,001
Sim	148 (36,0)	71 (48,0)	77 (52,0)	
Não	263 (64,0)	69 (26,2)	194 (73,8)	
Características infantis				
Idade da criança				0,011
< 6 meses	44 (10,7)	22 (50,0)	22 (50,0)	
6 meses a 2 anos	107 (26,0)	42 (39,3)	65 (60,7)	
>2 anos a 6 anos	260 (63,3)	76 (29,2)	184 (70,8)	
Sexo da criança				0,034
Masculino	206 (50,1)	60 (29,1)	146 (70,9)	
Feminino	205 (49,9)	80 (39,0)	125 (61,0)	
Baixo peso ao nascer^b^				0,695
Sim	45 (10,9)	11 (24,5)	34 (75,5)	
Não	356 (86,6)	124 (34,8)	232 (65,2)	
Frequenta creche/pré-escola				0,107
Sim	190 (46,2)	57 (30,0)	133 (70,0)	
Não	221 (53,8)	83 (37,6)	138 (62,4)	

a) Teste qui-quadrado de Pearson; Teste exato de Fisher; b) Não foi possível obter informações para a totalidade da amostra.

A Tabela 2 apresenta a comparação das características entre os grupos de mães beneficiárias e não beneficiárias do Programa Bolsa Família, de acordo com o uso e o preenchimento da caderneta da criança. Observou-se maior proporção de uso da caderneta entre as mães beneficiárias do programa, com diferença significativa: 70% possuíam a caderneta (p < 0,001), 63,1% estavam com a caderneta em mãos (p = 0,037) e 65,8% já tinham lido completamente a caderneta (p < 0,001). Quanto ao preenchimento, das 282 mães que estavam com as cadernetas em mãos no momento da entrevista, apenas 32,9% das cadernetas apresentavam o preenchimento completo das curvas de crescimento (p = 0,579); 25,2% tinham preenchimento completo da ficha de desenvolvimento infantil (p = 0,101); e 85,8% estavam com o calendário vacinal completo (p = 0,005).

**Tabela 2:** Distribuição das variáveis relativas ao uso e ao preenchimento da caderneta da criança entre mães e crianças beneficiárias e não beneficiárias do Programa Bolsa Família, Salvador, Bahia, 2023

Variáveis		Beneficiárias do Programa Bolsa Família		
	Total	Não (n = 140)	Sim (n = 271)	p-valor^a^
	n (%)	n (%)	n (%)	
A criança possui a caderneta da criança do Ministério da Saúde?				< 0,001
Sim	337 (82,0)	101 (30,0)	236 (70,0)	
Não	74 (28,0)	39 (52,7)	35 (47,3)	
Está com a caderneta da criança em mãos?				0,037
Sim	282 (68,6)	104 (36,9)	178 (63,1)	
Não	129 (31,4)	36 (27,9)	93 (72,1)	
A senhora já leu a caderneta da criança?				< 0,001
Completamente	73 (17,8)	25 (34,2)	48 (65,8)	
Parcialmente	203 (49,4)	71 (35,0)	132 (65,0)	
Nunca li	106 (25,8)	25 (23,6)	81 (76,4)	
Não tenho	29 (7,0)	19 (65,5)	10 (34,5)	
Preenchimento das curvas de crescimento^b^				0,579
Sim	93 (32,9)	33 (35,5)	60 (64,5)	
Não	74 (26,2)	31 (41,9)	43 (58,1)	
Incompleto	115 (27,9)	40 (34,8)	75 (65,2)	
Preenchimento da ficha do desenvolvimento infantil^b^				0,101
Sim	71 (25,2)	24 (33,8)	47 (66,2)	
Não	113 (40,1)	50 (44,2)	63 (55,8)	
Incompleto	98 (34,7)	30 (30,6)	68 (69,4)	
Completude do calendário vacina^b^				0,005
Sim	242 (85,8)	80 (33,1)	162 (66,9)	
Não	40 (14,2)	24 (60,0)	16 (40,0)	

a)Teste qui-quadrado de Pearson; b) Apenas das cadernetas que estavam em mãos no momento da entrevista (n=282).

Apesar de não haver diferença significativa, a avaliação do preenchimento da caderneta da criança por faixa etária, entre as crianças beneficiárias do Programa Bolsa Família, apontou maior proporção de preenchimento das curvas de crescimento (57,1%), ficha de avaliação do desenvolvimento infantil (35,7%) e completude vacinal (92,7%) entre as crianças menores de 6 meses de idade, conforme a Tabela 3.

**Tabela 3:** Distribuição das crianças beneficiárias do Programa Bolsa Família com a caderneta da criança, no momento da entrevista, de acordo com a faixa etária e o preenchimento, Salvador, Bahia, 2023

Faixa etária		Preenchimento das curvas de crescimento				Preenchimento da ficha de desenvolvimento infantil				Completude do calendário vacinal		
	Total	Sim	Não	Parcial	p-valor^a^	Sim	Não	Parcial	p-valor^a^	Sim	Não	p-valor ^a^
	n	n (%)	n (%)	n (%)		n (%)	n (%)	n (%)		n (%)	n (%)	
< 6 meses	14 (7,9)	08 (57,1)	04 (28,6)	02 (14,3)	0,137	5 (35,7)	5 (35,7)	4 (28,6)	0,927	13 (92,7)	01 (7,1)	0,927
6 meses - < 2 anos	46 (25,8)	16 (34,8)	13 (28,2)	17 (37,0)		12 (26,1)	16 (34,8)	18 (39,1)		42 (91,3)	04 (8,7)	
> 2 anos - 6 anos	118 (66,3)	36 (30,5)	26 (22,0)	56 (47,5)		30 (25,4)	42 (35,6)	45(39,0)		107 (90,7)	11 (9,3)	
Total	178 (100,0)	60 (33,7)	43 (24,2)	75 (42,1)		47 (26,4)	63 (35,4)	68 (38,2)		162 (91,0)	16 (9,0)	

a) Teste qui-quadrado de Pearson; Teste exato de Fisher.

## DISCUSSÃO

Os resultados deste estudo mostraram que houve maior proporção de uso da caderneta da criança no grupo de mães beneficiárias do Programa Bolsa Família, com diferença estatisticamente significativa quando comparadas com o grupo de mães não beneficiárias. Quanto ao preenchimento das cadernetas, não houve diferença significativa entre os grupos, mas os resultados revelaram que as curvas de crescimento e as fichas de acompanhamento do desenvolvimento infantil tiveram maior proporção de registro no grupo de mães beneficiárias do programa, com o dobro de completude vacinal, quando comparadas com o grupo de mães sem o benefício. Entre as beneficiárias do Programa Bolsa Família, a maior proporção de preenchimento da caderneta da criança concentrou-se na faixa etária de zero a 6 meses de idade, apesar de não haver significância estatística.

Os achados sugerem que o grupo de mães beneficiárias do Programa Bolsa Família pode ter maior engajamento no uso da caderneta, e isso tem implicações significativas para a promoção da saúde infantil,^6^ mas que, infelizmente, os profissionais de saúde ainda não incorporaram o preenchimento da caderneta em sua prática como ferramenta de acompanhamento da saúde da criança. 

Algumas revisões da literatura, que incluíram estudos desde a década de 1990 até 2022, sobre a utilização da caderneta da criança no contexto brasileiro,^7^ ^),(^ ^9^ ^),(^ ^15^ corroboram os resultados obtidos, quando também apontam a subutilização da caderneta da criança pelos profissionais de saúde, justificada por problemas de infraestrutura e funcionamento dos serviços, tais como falta de tempo e capacitação dos profissionais, falta de equipamentos para antropometria das crianças e impressos para o registro.^16^ Acredita-se que esses aspectos afetam a adequada utilização da caderneta, e sua compreensão é crucial para a proposição de estratégias de enfrentamento desses problemas.

Adicionalmente, como agravante da baixa adesão ao preenchimento das curvas de crescimento e das fichas de acompanhamento do desenvolvimento infantil em ambos os grupos de mães, a distribuição da caderneta da criança não vem sendo realizada pelo Ministério da Saúde desde 2020, o que desmotiva ainda mais o seu uso pelos profissionais de saúde.^16^ Essa situação desrespeita as recomendações do Estatuto da Criança e do Adolescente quanto à proteção, garantia de saúde e responsabilidade social em relação às crianças.^17^

Entende-se que a condicionalidade de saúde preconizada pelo Programa Bolsa Família poderia incentivar maior uso e preenchimento da caderneta da criança, pois promove a participação ativa das famílias nos serviços de saúde, fomentando a responsabilidade parental, bem como proporciona um acompanhamento mais regular da criança nos serviços, facilitando o acesso a cuidados preventivos e de promoção à saúde na primeira infância de famílias em maior vulnerabilidade social.^18^

De fato, os resultados são animadores quando se comparam mães beneficiárias e não beneficiárias do Programa Bolsa Família, mostrando que as primeiras tiveram maior uso e preenchimento das cadernetas, em acordo com a hipótese deste estudo. No entanto, essa proporção ainda está aquém do esperado, considerando-se que isso ocorreu em apenas, aproximadamente, um terço da amostra. Uma revisão de escopo, que avaliou estudos brasileiros dos anos de 1990 até 2022, mostrou que o preenchimento das curvas de crescimento pode variar entre 8,9% e 100%; o das fichas de acompanhamento do desenvolvimento infantil varia entre 0% e 72,7%; e a variação da completude vacinal está entre 91,8% e 100%.^9^

A existência de condicionalidades no Programa Bolsa Família tem o propósito de favorecer o acesso a direitos sociais básicos de saúde e educação, com vistas a se romper o ciclo intergeracional da pobreza nas famílias beneficiárias.^19^ Nesse sentido, vale destacar o perfil sociodemográfico das mães que participaram do estudo. As jovens mães pardas e pretas são as mais vulneráveis nessa sociedade, já que experimentam desigualdades de raça, gênero e classe, bem como desvantagens de escolaridade e renda, vivem em condições de moradia precárias e, ao mesmo tempo, têm mais filhos e menos parceiros fixos.^20^ É fato a relação direta entre a extrema vulnerabilidade social e os impactos na saúde e no acesso aos serviços, com desdobramentos negativos para o cuidado com as crianças. 

Estudo realizado com uma coorte de 100 milhões de brasileiros cadastrados no Cadastro Único para Programas Sociais do Governo Federal (CadÚnico) analisou 6.309.366 crianças menores de 5 anos entre 2006 e 2015.^21^ Os resultados mostraram que o Programa Bolsa Família impactou positivamente para as melhorias de saúde, por meio do aumento do poder de compra, melhoria ao acesso na educação infantil e adesão aos programas de saúde para crianças, com a redução de 17% das chance de mortes.^21^

O Programa Bolsa Família enfatiza a oferta de ações que já fazem parte da linha de cuidado à saúde da criança nos serviços da APS.^22^ No entanto, observa-se que essas ações acabam se resumindo a tarefas meramente administrativas, de preenchimento de planilhas, sem que haja, na maioria das vezes, uma avaliação do profissional de saúde que possa garantir um cuidado integral da saúde dessas crianças.^18^

Estudo que buscou compreender as narrativas dos profissionais da APS do município do Rio de Janeiro, entre 2018 e 2019, identificou que, na prática, o acompanhamento das condicionalidades de saúde do Programa Bolsa Família é reduzido a “pesar e medir” as crianças, sem que necessariamente se realize um diagnóstico nutricional para a identificação dos casos de risco e intervenções oportunas.^18^ É preocupante a visão burocrática dessas condicionalidades de saúde, apenas para garantir que a família não perca o benefício e que as metas de gestão do Programa Bolsa Família sejam cumpridas, pois o comparecimento da criança para a coleta de dados do programa deveria ser oportunidade de orientação, educação em saúde, avaliação da criança e preenchimento da caderneta da criança. Porém, percebe-se pouca valorização desse momento de comparecimento das crianças à unidade de saúde.^23^ ^)^

Por outro lado, é crucial avançar na reorganização dos serviços de saúde e no engajamento e sensibilização dos profissionais, para se garantir que as intervenções de saúde e sociais sejam oferecidas de maneira integrada e com excelência para todas as famílias com crianças na primeira infância.^3^ ^),(^ ^19^ Isso implica promover uma abordagem integral, que considere não apenas as necessidades de saúde imediatas, mas também as questões sociais e econômicas que impactam o bem-estar das famílias, integrando setores como saúde, educação, assistência social e desenvolvimento comunitário.^3^

Este estudo foi realizado em um único momento e local, limitando o estabelecimento de relações causais em uma sequência temporal, o que pode se constituir em uma limitação. No entanto, apresentam-se dados ainda pouco explorados sobre o uso e o preenchimento da caderneta e sua relação com o Programa Bolsa Família.

Conclui-se que os beneficiários do Programa Bolsa Família apresentam maior uso e preenchimento da caderneta da criança. No entanto, é importante ressaltar que esse preenchimento ainda não atende completamente às recomendações dos órgãos nacionais de saúde. Diante disso, torna-se necessário implementar ações de educação permanente em serviço, sensibilização e empoderamento das famílias, tais como campanhas de conscientização e educação em saúde, incluindo materiais educativos, palestras em unidades de saúde e atividades, além do incentivo da corresponsabilização nos territórios, via lideranças comunitárias. Essas medidas são essenciais para que a caderneta possa desempenhar efetivamente seu papel como um instrumento de acompanhamento do crescimento e desenvolvimento de todas as crianças, promovendo, assim, uma melhoria significativa nos indicadores da saúde infantil.
